# Real world effectiveness of Mycophenolate-sodium therapy in patients at risk with Graves’ orbitopathy

**DOI:** 10.1186/s13044-025-00263-6

**Published:** 2025-10-01

**Authors:** Julius Sander, Karim Al-Ghazzawi, Nikolaos Bechrakis, Ying Chen, Inga Neumann, Anja Eckstein, Michael Oeverhaus

**Affiliations:** 1 Department of Ophthalmology, University Hospital Essen, Hufelandstr. 55, 45147 Essen, Germany; 2 Medical practice Dres. Oeverhaus, Rietberg, Germany

**Keywords:** Thyroid eye disease, GO, TED, MPS

## Abstract

**Purpose:**

Patients with active, moderate-to-severe Graves’ orbitopathy require immunosuppressive treatments to reduce inflammation and morbidity. Since 2021 EUGOGO lists Mycophenolate-sodium (MPS) as first-line-treatment, which lead to a change in treatment regimens. In our center MPS was administered mainly for patients at risk for deterioration (e.g. unstable thyroid function, smoker etc.) or as second-line treatment. To augment the limited data we analyzed our real-world cohort retrospectively.

**Methods:**

We analyzed all consecutive patients of our tertiary referral center (2019–2023) with a complete data set, who either received MPS simultaneously with intravenous methylprednisolone (IVMP), or after a first course of IVMP.

**Results:**

We evaluated the data of 172 patients. Ninety-five were eligible for analysis. Clinical Activity Score showed a significant decrease between baseline (BL) and primary endpoint 6 months (3.9 ± 0.9 vs. 2.4 ± 1.4, *p* < 0.0001). Inactivation was achieved in 60% of all patients at 6 months and in 77% at 12 months. Deviation, motility, upper eye lid retraction and proptosis showed no significant changes after 6 months. TSH-receptor-antibody-levels (TRAb) showed a significant decrease at 3 and 6 months (*p* < 0.0001). 10.5% developed DON. Multiple logistic regression showed a significant influence of irradiation after BL for inactivation (OR 6.18, 95% CI: 1.08 to 48.99).

**Discussion:**

While inactivation is most often achieved, the severity of the disease in form of fibrosis (lid retraction, motility) and proptosis is not reversed. Further rehabilitative surgery is needed and patients should still be closely monitored for DON. Other immunosuppressants could be more effective even in IVMP resistant GO and should be subject to randomized head-to-head trials.

**Supplementary Information:**

The online version contains supplementary material available at 10.1186/s13044-025-00263-6.

## Introduction

Graves’ orbitopathy (GO) is an autoimmune disease and the most common extrathyroidal manifestation of Graves’ disease (GD) [1, 2, 3, 4]. It occurs when TSH receptor autoantibodies (TRAb) stimulate receptors on orbital fibroblasts, which triggers inflammatory and proliferative cascades [5, 6]. Patients experience symptoms of soft tissue inflammation, extraocular muscle fibrosis (diplopia, lid retraction), and varying degrees of proptosis, which can significantly affect their quality of life [7, 8]. In severe cases, the disease may threaten vision, primarily due to optic nerve compression [9]. Treating severe GO remains challenging. Until recently, glucocorticoids were the cornerstone of treatment, with the most common first-line regimen for active moderate-to-severe GO being weekly intravenous methylprednisolone (IVMP) injections over 12 weeks [10]. However, the treatment landscape has changed considerably due to the development of new therapies: Anti-IL-6 treatments, IGF1R-Blockage, statins, sirolimus and other new options have emerged. In the moment there are two treatment guidelines available, one from ATA/ETA (2022) and one from EUGOGO (2021) [10, 11]. The recommendations differ significantly, which is partly due to the limited availability, especially of the new IGF1-Receptor-Blocker Teprotumumab (only in USA, Brazil, Japan). Thus, in Europe the 2021 EUGOGO guideline is still the first choice. In this guideline Mycophenolate-sodium (MPS, 2 × 360 mg for 24 weeks) is recommended in combination with IVMP for 12 weeks (4.5 g) as the first-line treatment, rather than positioning MPS as a second-line therapy as in previous guidelines [10, 12]. This is done according to the results of a RCT published 2018 where it was shown that addition of MPS to treatment with IVMP improved the rate of treatment response at 24 weeks [13]. Most other clinical trials have utilized Mycophenolate-Mofetil, an ester prodrug of the active compound mycophenolic acid (MPA), which has shown similar efficacy and safety profiles to MPS [14, 15, 16]. MPA exerts its effects by reducing the production of guanosine triphosphate, thereby suppressing purine synthesis and preventing the proliferation of activated T lymphocytes, ultimately leading to their apoptosis. MPS, formulated in an enteric-coated capsule, is thought to have a lower incidence of gastrointestinal side effects by delaying the release of MPA until it reaches the small intestine [17, 18]. However, data on the efficacy and safety of MPS in GO is limited to 213 patients from the aforementioned RCT and an earlier prospective single-centre trial [13, 16]. Therefore, our objective was to retrospectively collect real-world data on the combination therapy of MPS with IVMP. In contrast to the aforementioned RCT we focussed on MPS as first-line therapy in risk patients and as a second-line treatment in relapsing or resistant cases following IVMP.

## Patients and methods

### Study population

**Fig. 1:**
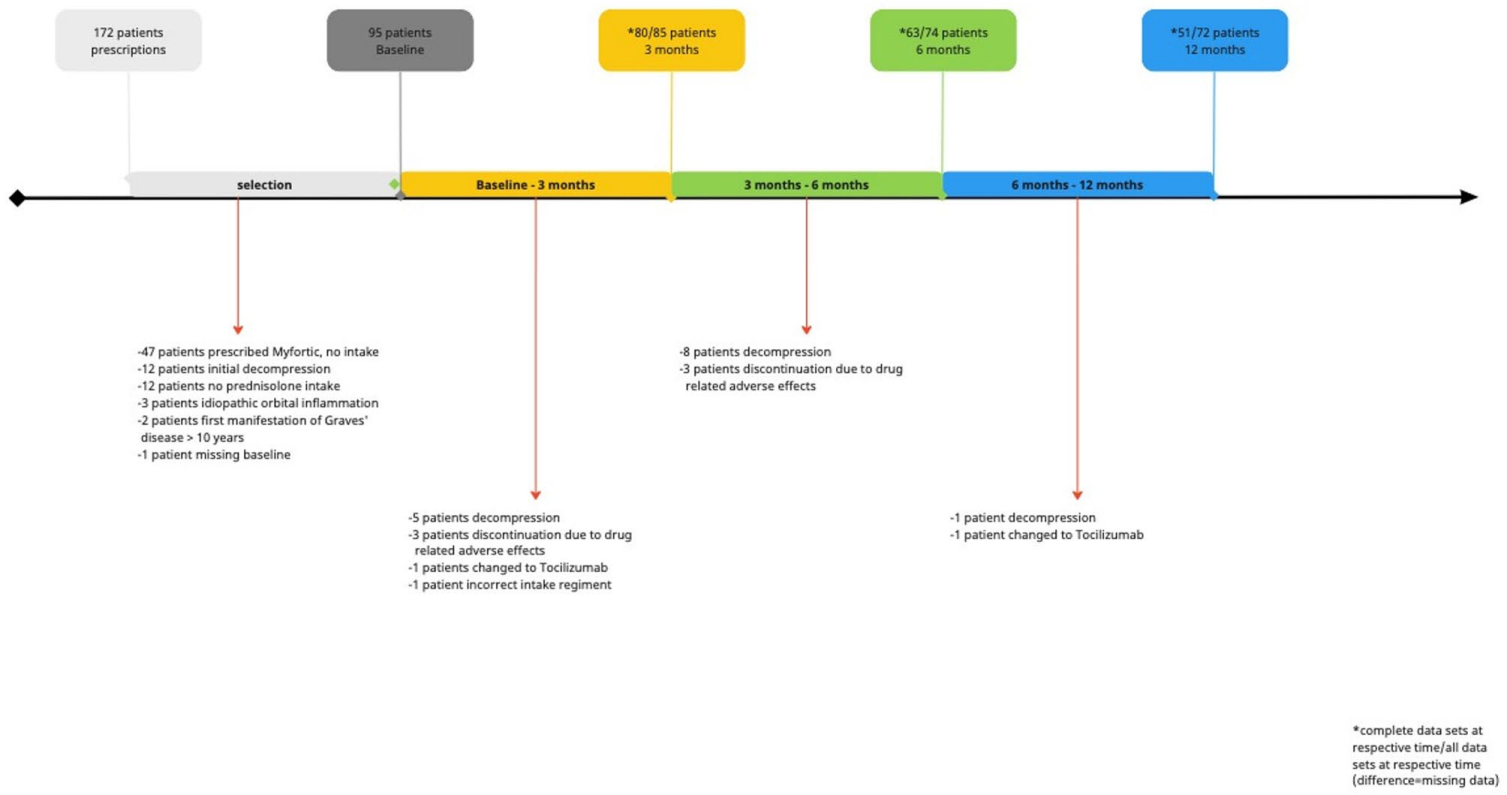
Study cohort at baseline and follow-up timepoints

We collected the clinical and demographic data of all consecutive patients of our tertiary referral center from April 2019 till March 2023 and analyzed it with descriptive statistics. Only patients with an actual diagnosis of GO and a complete data set were included in the further analysis. Baseline characteristics, clinical presentation and the course of the disease (treatments, surgeries) were assessed. From this previously published database of 4260 patients (GODE: Graves’ orbitopathy Database Essen) [19, 20], we selected the patients with MPS prescription (*n* = 172). For analysis we focused on patients who received both MPS and IVMP, had an initial moderate-to-severe GO and a first manifestation no longer than 5 years ago. This resulted in an analyzable study population of *n* = 95 (see Fig. 1 for reasons of exclusion, noteworthy: 47 patients were prescribed but did not take MPS). Baseline characteristics and the course of the disease (treatments, surgeries) were assessed based on the patients’ charts. Due to limited data, MPS was prescribed in our institution mainly for more severe cases, whereas IVMP treatment was still first choice for most moderate to severe cases even after the 2021 EUGOGO guideline [10]. All patients received first 6 × 500 mg IVMP. After that they either received MPS with another 6 × 250 mg or 6 × 500 mg IVMP. If the patients already had 12 × 500 mg IVMP an still presented with relapsing or resistant GO we used MPS as monotherapy. All patients received 2 × 360 mg MPS daily orally. The retrospective study was performed in accordance with the Declaration of Helsinki and approved by the Ethics Commission of the University of Essen (reference number: 22-10729-BO). All data were de-identified, and the requirement for informed consent was waived due to the retrospective nature of the study.

### Clinical assessment

All patients were assessed by a team comprising specialized ophthalmologists (AE, MO, YC, IN) and a skilled orthoptist. The diagnosis of GO was established by identifying typical clinical signs during the examination, which included measurements of BCVA, slit-lamp biomicroscopy, applanation tonometry, funduscopy, Hertel exophthalmometry, assessment of subjective diplopia and objective measurement of deviation (far and near distance) using the prism cover test and measurement of monocular excursions with Kestenbaum glasses [21]. Thyroid disease was categorized into Graves’ disease (active hyperthyroidism or already treated), primary hypothyroidism and euthyroidism (no thyroid disease in follow-up examinations). In the absence of thyroid disease we utilized clinical signs, MRI or CT images and levels of thyroid-specific antibodies (TRAb, Anti-TPO) to diagnose euthyroid GO. The TRAb Elecsys assay (Cobas, Roche), which has a detection limit of 0.3 IU/l according to the package inserts, was used as standard in the clinical routine [22]. The activity of GO was assessed using the Clinical Activity Score (CAS) and our previously described **Soft Tissue Score (STS)** classification systems, which is derived from the CAS but scores more gradually and both eyes as follows [23, 24]: spontaneous retrobulbar pain (0–1), painful eye movement (0–1), lid redness (0–1), conjunctival injection (0–1), lid edema (0–1), swelling caruncle (0–1) and chemosis (0–1). The STS score-derived signs of soft tissue inflammation were assessed as follows: spontaneous retrobulbar pain (0–1), painful eye movement (0–1), upper lid edema (0–2), lower lid edema (0–2), conjunctival injection (0–1), chemosis (0–1), lid redness (0–1) and swelling of caruncle or plica (0–1). The sum builds the clinical soft tissue score (STS).

A CAS score of ≥ 3/7 indicated active GO. Furthermore, we classified GO severity as mild, moderate-to-severe or sight-threatening (dysthyroid optic neuropathy (DON) and/or corneal breakdown) according to the EUGOGO criteria [12].

### Statistical evaluation

To analyze metric data, median values (*x*) and range or mean and standard deviation (SD) were computed. An ordinary one-way-ANOVA was used to assess differences between groups if the D’Agostino–Pearson omnibus normality test indicated normal distribution; otherwise, the Kruskal-Wallis-test was used. The paired t-test, if normally distributed, or the Wilcoxon matched-pairs signed rank test, if not normally distributed, was used to record the differences between two variables of the same measurement group. Fisher’s exact test was used to examine group distributions of binary variables. A level of statistical significance was defined as two-tailed with 2α < 0.05. All calculations were performed using Graph Pad Prism (Prism 10.3.1 for MacOS) *p*-values are provided descriptively with α-adjustment for multiple testing.

## Results

### Study population

Of the 95 patients, 38 received MPS + IVMP simultaneously while 57 patients received MPS for relapsing (*n* = 35) or resistant GO (*n* = 22) after IVMP therapy. Baseline characteristics are shown in Table 1. Since the groups did not differ significantly and showed similar results, treatment outcomes are reported in the following combined for all 95 patients to allow for easier reading (see Supplemental Fig. 1–3 for separated results).

**Table 1:** Characteristics of study population

	All (n = 95)
Age at onset^1^	54.0 ± 10.7
Treatment period^2^	6.3 ± 2.9
Time since onset^2;6^	13.1 ± 10.3
**Thyroid disease**	
Graves’ disease	89.5% (85/95)
Hypothyroidism	8.4% (8/95)
Euthyroidism	2.1% (2/95)
**Thyroid treatment**	
ATD	43.2% (41/95)
Thyroidectomy	31.6% (30/95)
Including prior thyroidectomy	54.7% (52/95)
Primary RAI	6.3% (6/95)
Including prior primary RAI	21.1% (20/95)
Orbital irradiation	
Including prior orbital irradiation	64.2% (61/95)
	72.6% (69/95)
**Lab results**	
TRAb	18.2 ± 16.4 (69/95)
fT3	5.4 ± 6.1 (71/95)
fT4	7.2 ± 13.7 (71/95)
TSH	3.0 ± 7.4 (76/95)
**Smoking status**	
Non-smoker	31.6% (30/95)
Smoker	34.7% (33/95)
Past smoker	33.7% (32/95)
Cigarettes per day	6.0 ± 9.0
**Activity markers**	
Proptosis^3^	20.5 ± 3.0 (94/95)
Motility^4^	294.4 ± 47.0
STS	4.4 ± 1.3 (93/95)
CAS	3.9 ± 0.9 (94/95)
Strabismus	52.6% (50/95)
Deviation vertical^5^	6.1 ± 11.2 (94/95)
Deviation horizontal^5^	2.4 ± 4.7 (93/95)
Upper eyelid retraction	44.2% (42/95)

Unless otherwise stated data are means ± SD or proportions (%) or median ($\:\stackrel{\sim}{x})$ [range]; a: t-test/ Mann-Whitney-test, b: Fishers exact test M + M und M + M < 4, c: ANOVA

Units: 1: years; 2: months; 3:mm; 4: degree; 5: PD; 6: of thyroid disease

Strabismus: includes patients with PD ≥ 2; Upper eyelid retraction includes patients with retraction ≥ 1 mm

Females were more frequent (*n* = 77) than men (*n* = 18), resulting in F/M ratio of 4.3. The mean age (SD) was 54.0 ± 11 (25–75) years. 89% of all 95 patients suffered from GD. 64% of patients received orbital irradiation during the acute treatment period. Overall, 73% did receive orbital irradiation. The thyroid treatment was structured as follows: 32% of the patient cohort underwent thyroidectomy during the acute treatment period (including prior thyroidectomy 55% overall), 43% received ATD and 6% underwent primary RAI immediately prior baseline (BL) (including prior primary RAI 21% overall). Overall, 68% of the study population smoked or were ex-smokers with an average number of cigarettes of 6.0 ± 9.0 per day (an overview of baseline characteristics is provided in Table 1).

### Inflammation

**Fig. 2:**
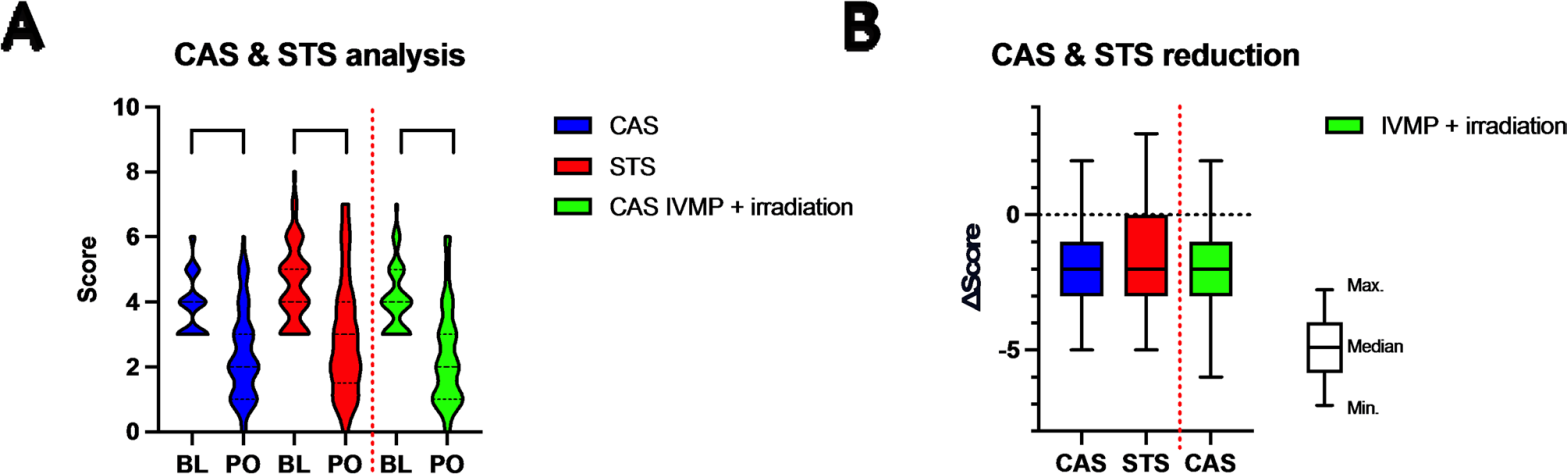
Inflammation: There was a significant reduction of Clinical Activity Score (CAS) and Soft Tissue Score (STS) after 6 months (**A**). The median reduction was − 2 for STS and − 2 for CAS in both groups. To compare the results to the classical intravenous methylprednisolone (IVMP) and irradiation treatment regime, we included a previously published study cohort in the analysis [24]. There was no significant difference between IVMP + Mycopenolate-sodium (MPS) and IVMP + irradation in terms of activity reduction (**B**). BL = Baseline; PO = Primary Outcome (6 months follow up); m = months; Minimum (Min.); Maximum (Max.) if not otherwise stated, then not significant; 0,05 > *p* > 0,001 ->*; 0,01 > *p* > 0,001 ->**; 0,001 > *p* > 0,0001 ->***; *p* < 0,0001 ->****

To look at the inflammation parameters in a bundled manner, we analyzed the CAS and Soft Tissue Score (STS), as well as their respective reduction after 6 months (Primary Outcome, PO). In the overall analysis of patients who received MPS and IVMP, there was a highly significant difference (3.9 ± 1 vs. 2.4 ± 1 *p* < 0.0001). STS analysis showed similar results (4.5 ± 1 vs. 2.9 ± 2 *p* < 0.0001). Both CAS and STS showed an average reduction in the CAS value of 1.5 ± 2 and an average STS reduction of 1.6 ± 2 (see Fig. 2). 30 patients showed a significant reduction of 2 points or more in CAS. Complete inactivation in terms of CAS (0–1) was observed in 17 patients. None of the individual components of CAS proved to be dominant. To compare the results to the classical IVMP and irradiation treatment regime, we included a previously published retrospective study cohort in the analysis (moderate-to-severe GO, *n* = 72) [24]. There was no significant difference between IVMP + MPS and IVMP + irradiation in terms of activity reduction (*p* = 0.4347). In order to compare patients who received IVMP + MPS with those who received radiation therapy in addition to IVMP + MPS, we performed another subgroup analysis. Patients who experienced adverse events during the treatment period and those who had previously undergone radiation therapy but not during the acute treatment period were excluded. In this analysis of the subgroups IVMP + MPS (*n* = 19) vs. IVMP + MPS + orbital irradiation (*n* = 49), 22 (45%) of the irradiated patients had a CAS < 3 after 6 months (12 (25%) patients had a CAS = 0–1)). Among patients who did not receive irradiation, 9 (47%) patients had a CAS < 3 (3 (16%) patients had a CAS = 0–1). There was no significant difference between IVMP + MPS and IVMP + MPS + orbital irradiation in terms of inactivation (CAS < 3 (*p* = 1.0); CAS = 0–1 (*p* = 0.5301)). Inactivation (CAS < 3) was found in 60% of the patient collective at 6 months. A further improvement in the inactivation rate was seen after additional 6 months. Here, 77% of the patient collective was inactive at 12 months follow-up. Of note, 54% of patients required treatment with MPS for longer than the EUGOGO recommendation of 6 months, due to persisting CAS ≥ 3 or progressing changes in severity [10]. On average, patients took MPS for 6.3 ± 3 months. The longest duration of MPS therapy was 13.6 months.

## Severity

**Fig. 3:**
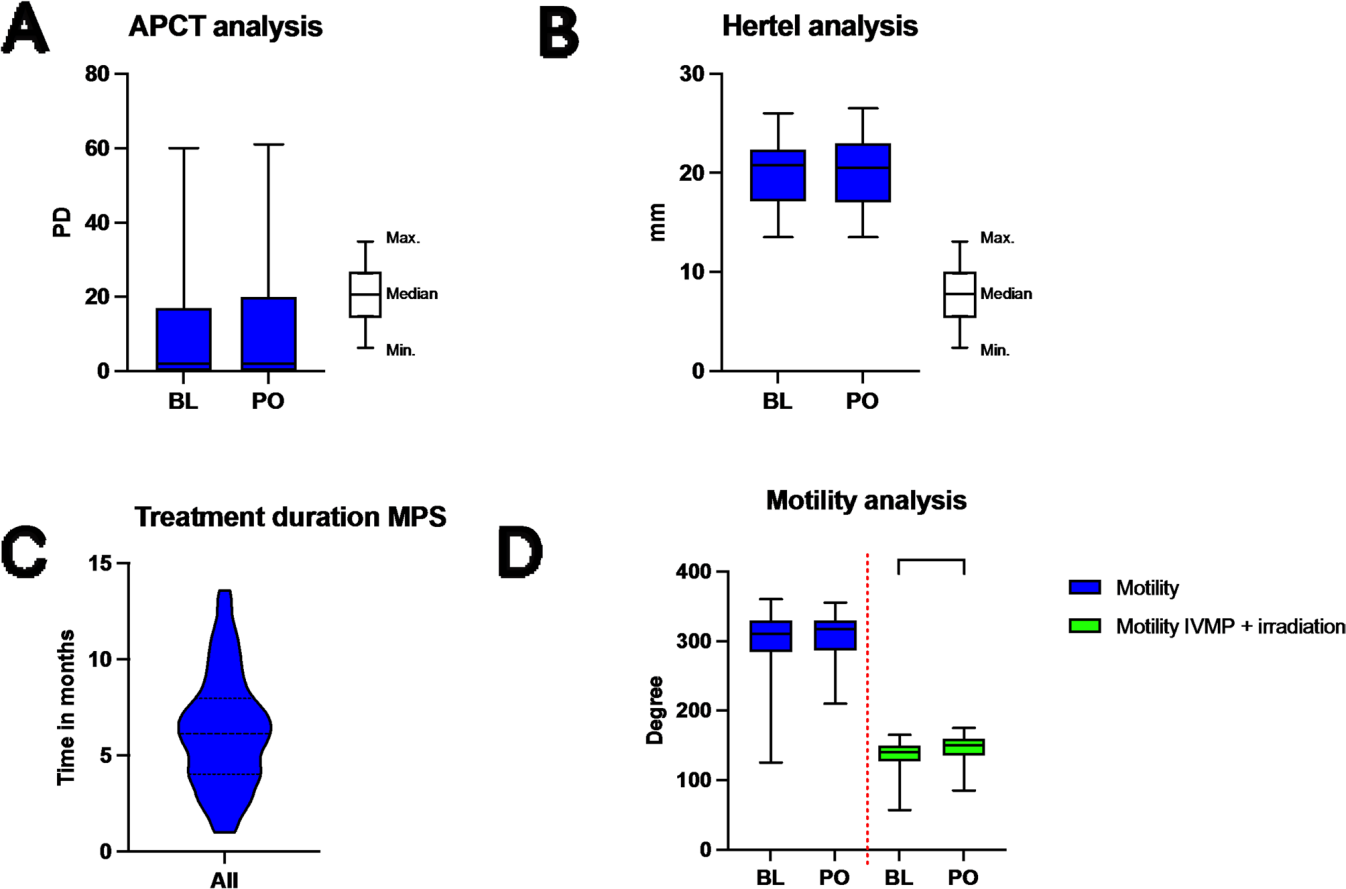
Severity: The combined amount of deviation (APCT) did not change significantly after treatment (**A**). Same was true for proptosis (**B**). Violin plot of treatment duration: Mean: 6.27 months, Std. Deviation: 2.93 months (**C**). Motility has not changed significantly either. To compare the results to the classical IVMP and irradiation treatment regime, we included a previously published study cohort in the analysis [24]. Motility changed significantly in the IVMP + irradiation cohort. (**D**). BL = Baseline; PO = Primary Outcome (6 months follow up); m = months; Minimum (Min.); Maximum (Max.); Mycopenolate-sodium (MPS); All includes all patients with MPS; if not otherwise stated, then not significant; 0,05 > *p* > 0,001 ->*; 0,01 > *p* > 0,001 ->**; 0,001 > *p* > 0,0001 ->***; *p* < 0,0001 ->****

There were no significant differences in the severity parameters, comparing baseline population to primary outcome (see Fig. 3). In the alternating prism cover test (APCT), there were no significant differences between the two time points. There were also no significant differences in the proptosis analysis (20.1 ± 3 vs. 20.2 ± 3 *p* = 0.83) and upper eye lid retraction (0.7 ± 1 vs. 0.8 ± 1 *p* = 0.21). Only two patients showed a significant reduction in proptosis of ≥ 2 mm. The findings for upper eyelid retraction were also stable. Two patients showed a significant reduction in upper eyelid retraction of ≥ 1 mm. A slight improvement in the average motility values with IVMP + MPS therapy was noted, but this was not statistically significant (299.2 ± 43 vs. 304.8 ± 34 *p* = 0.11). The subgroub analysis between IVMP + MPS and IVMP + MPS + orbital irradiation showed that 16 (33%) of the irradiated patients improved by > 8° in motility after 6 months. Among the non-irradiated patients, 3 (16%) patients improved significantly. There was no significant difference between IVMP + MPS and IVMP + MPS + orbital irradiation in terms of motility (*p* = 0.2322). 10.5% developed DON during the observation period despite MPS. Irradiation had no significant effect on occurrence of DON with 9.8% despite irradiation vs. 11.8% without.

## Biochemical markers

**Fig. 4:**
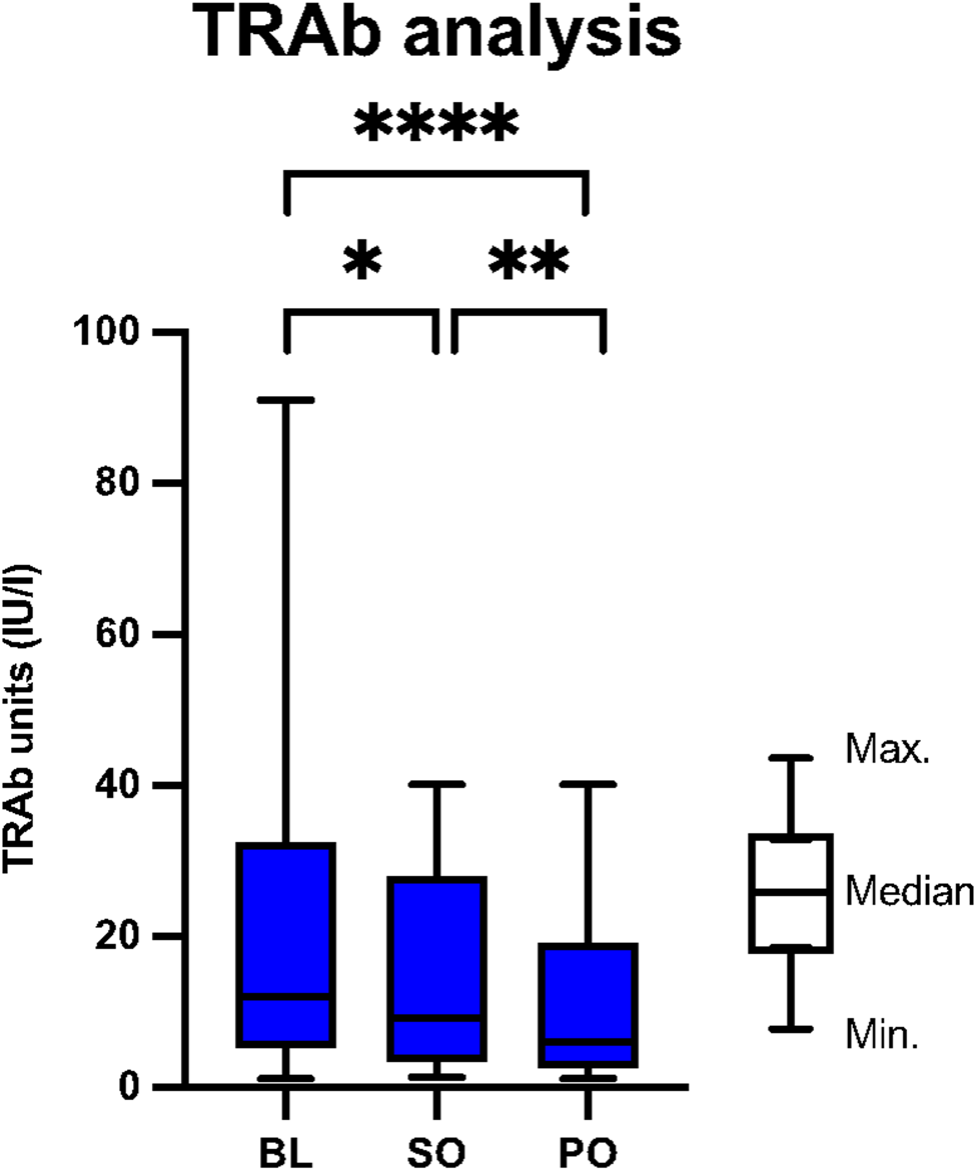
Biochemical markers: The antibody level against TSH-Receptor (TRAb) changed significantly in all groups. BL = Baseline; PO = Primary Outcome (6 months follow up); m = months; Minimum (Min.); Maximum (Max.); SO = Secondary Outcome (3 months follow up); if not otherwise stated, then not significant; 0,05 > *p* > 0,001 ->*; 0,01 > *p* > 0,001 ->**; 0,001 > *p* > 0,0001 ->***; *p* < 0,0001 ->****

In the separate analysis of the biochemical markers, we paid particular attention to the TRAb analysis. Only complete data sets were included here. As previously mentioned, the analysis showed significant reduction in TRAb levels either after 3 or after 6 months, with a highly significant reduction in the biochemical marker (18.2 vs. 12.1 *p* < 0.0001) between BL and PO (see Fig. 4). The average TRAb reduction amounted to 6.1 IU, which represents a percentage decrease of 67%. Five patients showed immunological remission defined as normal values indicated by the TRAb Elecsys assay (Cobas, Roche) [22]. In order to present an adjusted analysis and exclude any confounders, we again analyzed all patients who did not receive Tx or RAI around and after BL separately. For this purpose, we separated all patients who received such therapy during the treatment period or up to 6 months before BL (see Table 1 for details). Here, there was also a highly significant reduction in TRAb values (13.6 vs. 7.5 *p* < 0.0001) between BL and PO. However, there was no statistically significant reduction observed between BL and secondary outcome (SO), nor between SO and PO.

### Multiple logistic regression

**Fig. 5:**
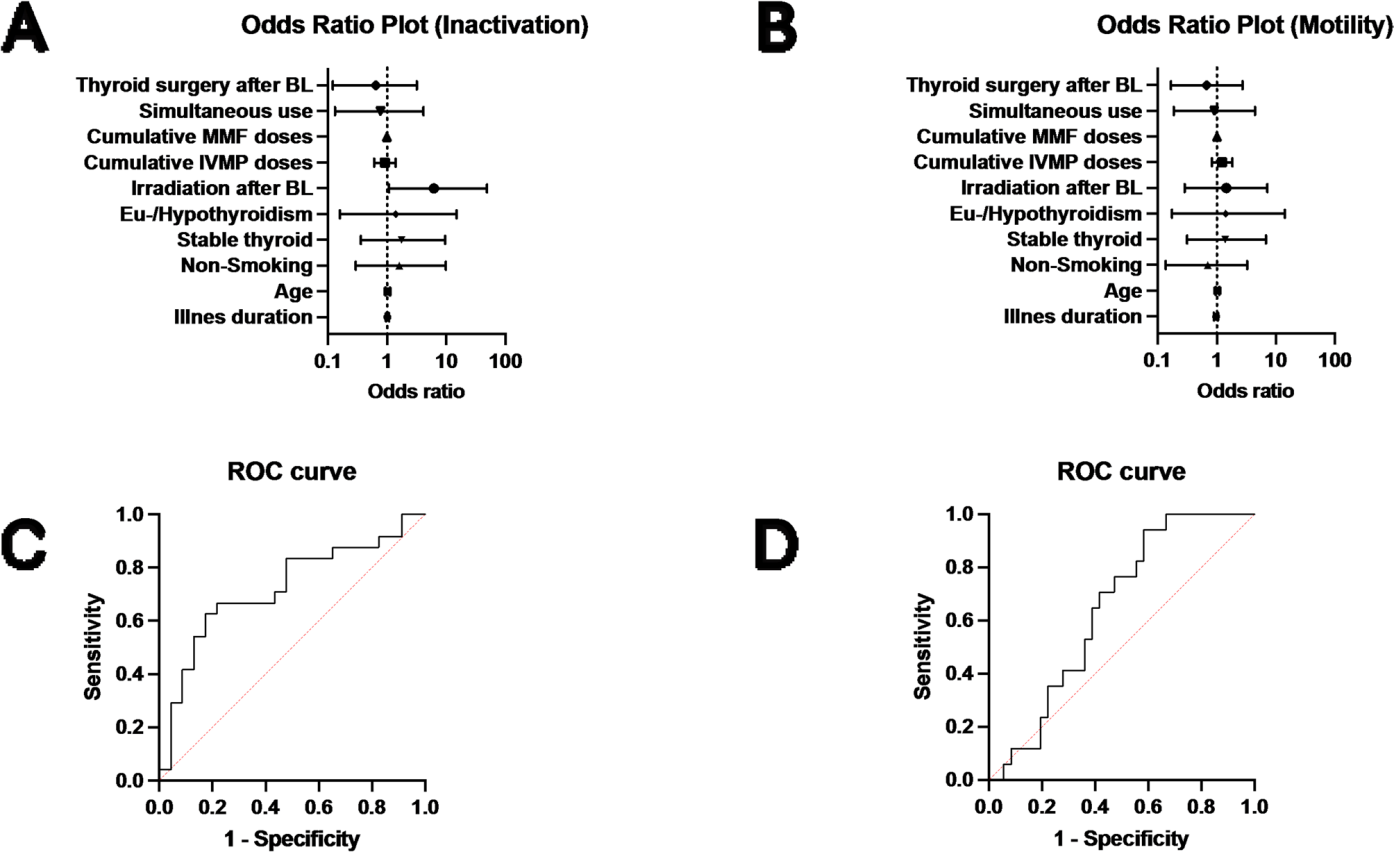
Multiple logistic regression: The multiple logistic regression showed a significant influence of irradiation after baseline (BL) for inactivation (**A**). Motility showed a correlation with several factors, which were not statistically significant (**B**). Corresponding Receiver-operating characteristic curve (ROC) graphs are presented in (**C**) and (**D**) displaying the limitations of the models. Mycophenolate mofetil (MMF); Intravenous methylprednisolone (IVMP)

We carried out a multiple logistic regression to determine the influence of individual variables on the inactivation (CAS < 3): the model incorporated, aside from illness duration, age of patients, non-smoking, stable thyroid, eu-/hypothyroidism, irradiation, cumulative IVMP doses, cumulative MMF doses, simultaneous use of IVMP and MPS and thyroid surgery status. It showed that irradiation after BL (OR 6.18, 95% CI: 1.08 to 48.99) had a considerable influence on inactivation (see Fig. 5). In contrast, illness duration (OR 1.01, 95% CI: 0.95 to 1.08), age of patients (OR 1.02, 95% CI: 0.94 to 1.12), cumulative IVMP doses (OR 0.91, 95% CI: 0.60 to 1.39), cumulative MMF doses (OR 0.99, 95% CI: 0.98 to 1.01), simultaneous use of IVMP and MPS (OR 0.77, 95% CI: 0.13 to 4.12), thyroid surgery status (OR 0.64, 95% CI: 0.12 to 3.18), non-smoking (OR 1.6, 95% CI: 0.29 to 9.88), stable thyroid (OR 1.75, 95% CI: 0.36 to 9.56) and eu-/hypothyroidism (OR 1.40, 95% CI: 0.16 to 14.93) were not significantly associated. Multicollinearity analysis was employed to ensure the independence of the ten variables, which was the case. A goodness-of fit analysis showed a Nagelkerke’s R2 of 0.20 and a log-likelihood ratio (G²) of 7.80 (*p* = 0.64) indicating a good prediction model. The area under the receiver operating characteristic curve (AUC) was observed to be 0.72 (95% CI: 0.57–0.87), indicating a good separability. The positive predictive power was observed as 76% and the negative predictive power was observed as 69%.

## Discussion

The results of this retrospective cohort study of 95 patients of our tertiary referral centre showed that MPS is effective in treating high risk GO patients as a primary and secondary anti-inflammatory treatment in IVMP refractory patients. While, MPS mainly decreased the activity, the severity of the disease was not significantly affected.

### Study population

The patients analysed in this retrospective study were patients at risk for further deterioration (e.g. high TRAb, smoker, males, poor thyroid control) who received MPS as primary treatment or patients unresponsive to IVMP treatment and received MPS as a second line therapy. This is reflected in the baseline characteristics: Almost 70% of patients were current or former smokers. This is a strong contrast to the general GO population with about 60% of non-smokers [20]. As smoking is a well-known risk factor for exacerbation of GO, heavy smokers were selected for IVMP and MPS simultaneous therapy due to their high risk of deterioration. The at risk situation was also notable regarding the second risk factor, an unstable thyroid, resulting in more than 50% of our patients receiving a thyroidectomy (including prior thyroidectomy). For comparison only 34% of 4260 patients in our whole GO database needed a thyroidectomy [20]. Kahaly et al. reported even lower rates of thyroidectomy in their RCT with MPS (22–29%) [13]. Since euthyroidism was an inclusion criteria of that RCT, TRAb levels were also much lower compared to our study (7-8.9 vs. 18.2 IU/l). The mean age of our study population was also slightly higher than in the general GO population (54 vs. 50 years) representing a further risk factor [19, 20]. Interestingly, the gender distribution was not different, although male sex is known as a risk factor [10, 20, 25]. Another difference was that many of our patients had a time span of more than 6 months since onset of the disease. Naturally, especially relapsing and resistant cases, but also patients with prolonged diagnostic journeys. These patients were not treatment-naive, which is why a lower response rate to immunosuppressants must be assumed compared to the cohort by Kahaly et al. [13]. In comparison, the study by Rajabi et al. (2022) analyzing Mycophenolate-Mofetil with low dose oral prednisolone, found that in their selected cohort of 242 patients, 50% of patients had a time span of more than 6 months since the onset of the disease. In addition, the Rajabi et al. cohort did not include any patients who had received corticosteroid or immunosuppressive treatment for GO or other reasons within the previous 3 months [26].

Interestingly, 47 of 172 patients in our cohort prescribed with MPS did not take the drug. This might reflect a reluctance of long-term daily immunosuppression opposed to short-term weekly IVMP infusions and fear of possible side effects (e.g. infections). A possible influential factor to this observation might be the COVID19 pandemic, which was partly during the observation period, possibly resulting in higher fear of infections. Six patients showed adverse drug effects and one patient who showed an incorrect intake pattern of MPS and had to be excluded from analysis. In our cohort, gastrointestinal side effects and, in some cases, flu-like infections were most common. This is consistent with the most common side effects reported by Kahaly et al. Other frequently observed side effects such as general disorders and administration side reactions (e.g., fatigue) or psychiatric disorders such as sleeping disorders were not observed in our cohort [13].

A further interesting observation was that in our patient cohort, a longer duration of therapy was needed opposed to the 24 weeks recommended by the guidelines [10]. Although the average duration of therapy was only slightly longer than the recommended time (6.3 vs. 6 months), the longest duration of treatment was just over a year. The duration was adapted individually to personal risk factors and treatment outcomes, as recommended to achieve a more personalized, tailored medicine. Patients should be informed, that therapy with MPS is a long term commitment to manage expectations for rapid improvement of inflammation.

### Inflammation

As in previous RCT studies analysing MPS, inflammation could be effectively reduced even in this real-world at risk population when given simultaneously with IVMP [13]. In contrast to the aforementioned RCT we did not have an IVMP group as direct comparison, but similar to the mentioned RCT by Kahaly et al., we did not find a superiority of adding MPS to IVMP when compared to our previously published IVMP and orbital irradiation cohort [13, 24]. Theoretically, one would expect due to the different immunosuppressive mechanisms of action of MPS and IVMP, that the administration of MPS has an additional effect in reducing inflammation. Additionally, it was also effective in reducing inflammation as a second line treatment in relapsing and resistant cases. 77% of our patient collective was inactive after 12 months (vs. 60% after 6 months). However, in reverse this means that even after 6 months of MPS as recommended by the EUGOGO and 12 weeks of IVMP 40% of these patients at risk and refractory patients were still active [10]. In the aforementioned study by Rajabi et al. treatment naïve cohort response rates were naturally higher with 90% activity improvement at 6 months [26]. In the study by Quah Qin Xian et al., which included a total of 20 patients, 8 patients showed moderate to severe GO. These patients received MPS after IVMP treatment. Here, clinical efficacy was achieved in all 8 patients after 6 months, but it must be emphasised that this is a very small patient cohort compared to our study. After 12 months, seven out of eight patients were still inactive [27]. In the study published by Li et al. (2023), a clinical effective rate of 83% was achieved with MPS and glucocorticoid pulse therapy after 6 months. 12-month values were not reported here. In comparison to our study, a combined score consisting of reduction of CAS, lid width, proptosis, diplopia and/or improvement in soft tissue involvement, which makes comparison with our study more difficult. Again, the number of patients receiving MPS and glucocorticoids was low, limited to 30 patients [28]. Furthermore, the comparison to our older cohort who received only IVMP and irradiation showed no significant superiority of MPS + IVMP treatment [24]. Therefore, other immunosuppressants might be considered instead of MPS for these severely afflicted patients. Of note, in a more stable patient population by Kahaly et al. inactivation was much higher at 6 months follow-up (80%) [13]. However, in the daily clinical setting patients are mostly not euthyroid when a treatment is required opposed to that RCT. Such less complicated cases were in our centre not selected for MPS treatment, but rather solely treated with IVMP with good effectiveness as shown in previous studies [29]. Further evidence of the severity of the disease in our cohort can be seen in comparison with the study by Shams et al. in which 0% of those treated with irradiation developed DON [30]. In our cohort, however, 9.8% developed DON despite irradiation. In contrast to most publications we compared the difference between patients receiving MPS and IVMP simultaneously or after evaluating the effect of IVMP following the first 6 weeks of infusions. Our data suggests that a first line therapy combination of MPS and IVMP in all patients is not mandatory, therefore the therapeutic effect of IVMP monotherapy can be evaluated first, before initiating a MPS therapy. Randomized controlled studies should be performed to further elucidate this topic and compare different immunosuppressants.

### Severity

Whether deviation (9 vs. 9.8 PD), motility (299° vs. 305°), upper eye lid retraction (0.7 vs. 0.8) nor proptosis (20 vs. 20 mm) showed significant changes after 6 months of MPS therapy. This should be discussed with the patients prior treatment to modulate their expectations for the treatment response which could otherwise lead to discontinuation of MPS since they see no more improvement. Our observation suggests that MPS does not have a significant effect on fibrosis and adipogenesis in GO patients, but only decreases Inflammation. Our findings regarding motility are in line with Kahaly et al. [13]. Other severity parameters were not reported separately, however integrated in their overall ophthalmic score, in which inflammation improvement was enough to count as overall improvement. Gorman diplopia scores were reported, but without analysis compared to baseline. Proptosis was reported by the authors as not reduced without numerical values. In contrast to our findings, the study by Quah Qin Xian et al. showed an improvement in the rather crude Gorman dipoplia score, but also an improvement in soft tissue involvement. Diplopia improved in 37.5% of patients after 6 and 12 months and worsened in only 12.5%. Proptosis and motility were not treated separately. Soft tissue involvement including pain and swelling was used to visualize soft tissue inflammation. Here too, 37.5% of patients showed an improvement in soft tissue involvement after both 6 and 12 months. Again, the small number of patients (*n* = 8) must be emphasized here [27]. The response rates were also higher in the treatment-naive cohort of Rajabi et al. with 83% proptosis and 88% diplopia improvement after 6 months [26]. In the study by Li et al. proptosis was listed separately for the right and left eye. After 6 months, the average value for the right eye was 17.6 ± 1 and for the left eye 18 ± 1 (vs. 20.1 ± 3 as the average value for both eyes). This showed a significant reduction compared to our cohort. Motility and diplopia were not mentioned separately in terms of numbers but were included in an overall score that showed an improvement compared to baseline [28]. Previously mentioned literature suggests that an improvement regarding severity of GO manifestation could be achieved in patients with less risk factors compared to our cohort. However, patients presenting at tertiary referral centers with a rather uncontrolled thyroid, high-risk patients e.g. smokers and therapy refractory cases might benefit from a more potent immunosuppressive treatment.

### Biochemical markers

The average percentage reduction in TRAb values was lower compared to the study by Lee et al., in which Tocilizumab was analyzed in GO patients (66.5% vs. 162.9%). In absolute values, however, the difference is not as pronounced (6.1 IU/L vs. 7.5 IU/L) [31]. We saw comparable results for the average absolute reduction in TRAb levels in the study by Habroosh et al. (6.1 IU/L vs. 7.81 IU/L). Here, too, the average reduction was slightly higher, albeit only to a small extent, when taking Tocilizumab [32]. In direct comparison with the study by Li et al., in which MPS and glucocorticoids were also used, there was a reduction in TRAb values from 17.4 ± 10 at baseline to 4.5 ± 3 after 6 months, which corresponds to an average reduction of 13 IU/l (vs. 6.1). However, only patients who had not received immunomodulatory therapy or cytotoxic drugs within the last 3 months were included in this study, whereas our study cohort included less treatment naïve patients [28]. Since our results are not obtained in a randomized controlled manner they should be interpreted with care, because other factors might have influenced the TRAb reduction. Due to the lack of a control group it is not possible to compare the results to IVMP and ATD/Thyroidectomy alone.

### Limitations

Due to the retrospective nature of this study, by far not all pressing questions could be answered and need further validation with randomized controlled studies. Interesting research questions of much earlier therapeutic effect of MPS on GO (preventive therapy with MPS) should be investigated, unfortunately once proliferative changes have taken place they do not seem to respond to immunosuppression. On the other hand positive effects especially on the long term prognosis of thyroid function could not be evaluated since most of the patient had already received definitive treatment of the thyroid. Here, we see a great potential since MPS significantly reduced TRAb levels which could lead to a better prognosis of the thyroid. Therefore, MPS might also be evaluated as a maintenance therapy after IGF1R blocking agents since that treatment does not influence TRAb levels, which might be responsible for relapses after IGF1R blocking treatments. It should be emphasized once again that the effect of IVMP monotherapy can be assessed before starting MPS therapy. Another interesting take away from our analysis is that some patients can benefit from a prolongated MPS treatment (more than the recommended 6 months) and that MPS can also be given as monotherapy in resistant/relapsing cases.

## Conclusion

The effect of MPS additional to IVMP as combination therapy for GO patients at risk or in IVMP non-responders as second line treatment is limited. Although inactivation is most often achieved, the severity of the disease in form of fibrosis of muscles (lid retraction, motility) and orbital volume increase (proptosis) is not reversed. Further rehabilitative surgery is needed, and patients should still be closely monitored for DON. Literature suggests that other biologicals/immunosuppressants seem to be more effective in treating activity and severity even in IVMP resistant GO and might be preferred over MPS. Randomized controlled studies should be performed to further elucidate this topic.

## Supplementary Information

Below is the link to the electronic supplementary material.

Supplementary Material 1

Supplementary Material 2

## Data Availability

No datasets were generated or analysed during the current study.
