# Supplementary material for: Real world effectiveness of Mycophenolate-sodium therapy in patients at risk with Graves’ orbitopathy
Source: Thyroid Res. 2025 Oct 1;18:46. doi: 10.1186/s13044-025-00263-6 (PMC12486492; doi:10.1186/s13044-025-00263-6)
Supplement: Supplementary file 1 — Supplementary Material 1 [file 13044_2025_263_MOESM1_ESM.docx]

|  | All (n=95) | | IVMP+MPS (n=38) | Resistant GO (n=22) | Relapsing GO (n=35) | *p* |  |
| --- | --- | --- | --- | --- | --- | --- | --- |
| Age at onset^1^  Treatment period^2^  Time since onset^2;6^ |  | 54.0 ± 10.7  6.3 ± 2.9  13.1 ± 10.3 | 52.1 ± 12.6  6.2 ± 2.7  8.9 ± 7.5 | 55.1 ± 9.0  6.5 ± 2.9  15.2 ± 13.0 | 55.5 ± 9.4  6.2 ± 3.3  16.3 ± 9.7 | 0.3343^c^  0.9144^c^  <0.0001^d^ |  |
| **Thyroid disease** |  |  |  |  |  |  |  |
| Graves’ disease |  | 89.5% (85/95) | 97.4% (37/38) | 90.9% (20/22) | 80% (28/35) | 0.5480^b^ |  |
| Hypothyroidism |  | 8.4% (8/95) | 2.6% (1/38) | 9.1% (2/22) | 14.3% (5/35) | 0.5480^b^ |  |
| Eutyhroidism |  | 2.1% (2/95) | 0% (0/38) | 0% (0/22) | 5.7% (2/35) | 1.0^b^ |  |
|  |  |  |  |  |  |  |  |
| **Thyroid treatment** |  |  |  |  |  |  |  |
| ATD |  | 43.2% (41/95) | 57.9% (22/38) | 50.0% (11/22) | 22.9% (8/35) | 0.5987^b^ |  |
| Thyroidectomy^7^ |  | 54.7% (52/95) | 52.6% (20/38) | 54.5% (12/22) | 57.1% (20/35) | 1.0^b^ |  |
| Primary RAI^7^  Orbital irradiation^7^ |  | 21.1% (20/95)  72.6% (69/95) | 18.4% (7/38)  71.1% (27/38) | 31.8% (7/22)  59.1% (13/22) | 17.1% (6/35)  82.9% (29/35) | 0.3428^b^  0.4010^b^ |  |
| **Lab results** |  |  |  |  |  |  |  |
| TRAb |  | 18.2 ± 16.4  (69/95) | 22.7 ± 20.1  (24/38) | 14.8 ± 13.0 (19/22) | 16.4 ± 14.4 (26/35) | 0.2180^d^ |  |
| fT3 |  | 5.4 ± 6.1  (71/95) | 6.7 ± 8.0 (28/38) | 6.5 ± 6.3 (17/22) | 3.4 ± 1.0 (26/35) | 0.0900^d^ |  |
| fT4 |  | 7.2 ± 13.7  (71/95) | 7.0 ± 17.6 (28/38) | 9.0 ± 14.6 (18/22) | 6.2 ± 6.9  (25/35) | 0.2730^d^ |  |
| TSH |  | 3.0 ± 7.4  (76/95) | 5.4 ± 10.1 (30/38) | 0.4 ± 0.7 (17/22) | 2.1 ± 5.7 (29/35) | 0.1585^d^ |  |
| **Smoking status** |  |  |  |  |  |  |  |
| Non-smoker |  | 31.6% (30/95) | 28.9% (11/38) | 50.0% (11/22) | 22.9% (8/35) | 0.1639^b^ |  |
| Smoker |  | 34.7% (33/95) | 42.1% (16/38) | 18.2% (4/22) | 37.1% (13/35) | 0.0881^b^ |  |
| Past smoker |  | 33.7% (32/95) | 28.9% (11/38) | 31.8% (7/22) | 40.0% (14/35) | 1.0^b^ |  |
| Cigarettes per day  **Activity markers**  Proptosis^3^  Motility^4^  STS  CAS  Strabismus  Deviation vertical^5^  Deviation horizontal^5^  Upper eyelid retraction |  | 6.0 ± 9.0  20.5 ± 3.0 (94/95)  294.4 ± 47.0  4.4 ± 1.3  (93/95)  3.9 ± 0.9  (94/95)  52.6% (50/95)  6.1 ± 11.2  (94/95)  2.4 ± 4.7  (93/95)  44.2% (42/95) | 6.4 ± 7.9  20.7 ± 3.1 (37/38)  293.1 ± 55.7  4.2 ± 1.1 (38/38)  3.8 ± 0.8  (38/38)  47.4% (18/38)  9.1 ± 13.8 (37/38)  1.4 ± 2.8 (37/38)  50.0% (19/38) | 3.0 ± 6.6  20.0 ± 2.7 (22/22)  307.3 ± 32.5  4.8 ± 1.4 (21/22)  3.8 ± 0.9  (21/22)  36.4% (8/22)  4.3 ± 11.1 (22/22)  0.9 ± 2.3 (22/22)  45.5% (10/22) | 7.3 ± 10.9  20.7 ± 3.2 (35/35)  287.7 ± 43.7  4.4 ± 1.4 (34/35)  4.0 ± 1.0  (35/35)  68.6% (24/35)  4.0 ± 6.9 (35/35)  4.4 ± 6.6 (34/35)  37.1% (13/35) | 0.1214^d^  0.6878^c^  0.2252^d^  0.2906^c^  0.7600^d^  0.4332^b^  0.3216^d^  0.0247^d^  0.7930^b^ |  |

**Supplemental Table 1:** Characteristics of study population

Unless otherwise stated data are means ±SD or proportions (%) or median ($\tilde{x})$ [range]; a: t-test/ Mann-Whitney-test, b: Fishers exact test M+M und M+M<4, c: ANOVA, d: Kruskal-Wallis test

Units: 1: years; 2: months; 3:mm; 4: degree; 5: PD; 6: of thyroid disease; 7: including prior treatment

Strabismus: includes patients with PD ≥2

Upper eyelid retraction: includes patients with retraction ≥1mm
