# Supplementary material for: Real world effectiveness of Mycophenolate-sodium therapy in patients at risk with Graves’ orbitopathy
Source: Thyroid Res. 2025 Oct 1;18:46. doi: 10.1186/s13044-025-00263-6 (PMC12486492; doi:10.1186/s13044-025-00263-6)
Supplement: Supplementary file 2 — Supplementary Material 2 [file 13044_2025_263_MOESM2_ESM.pptx]

## Slide 1
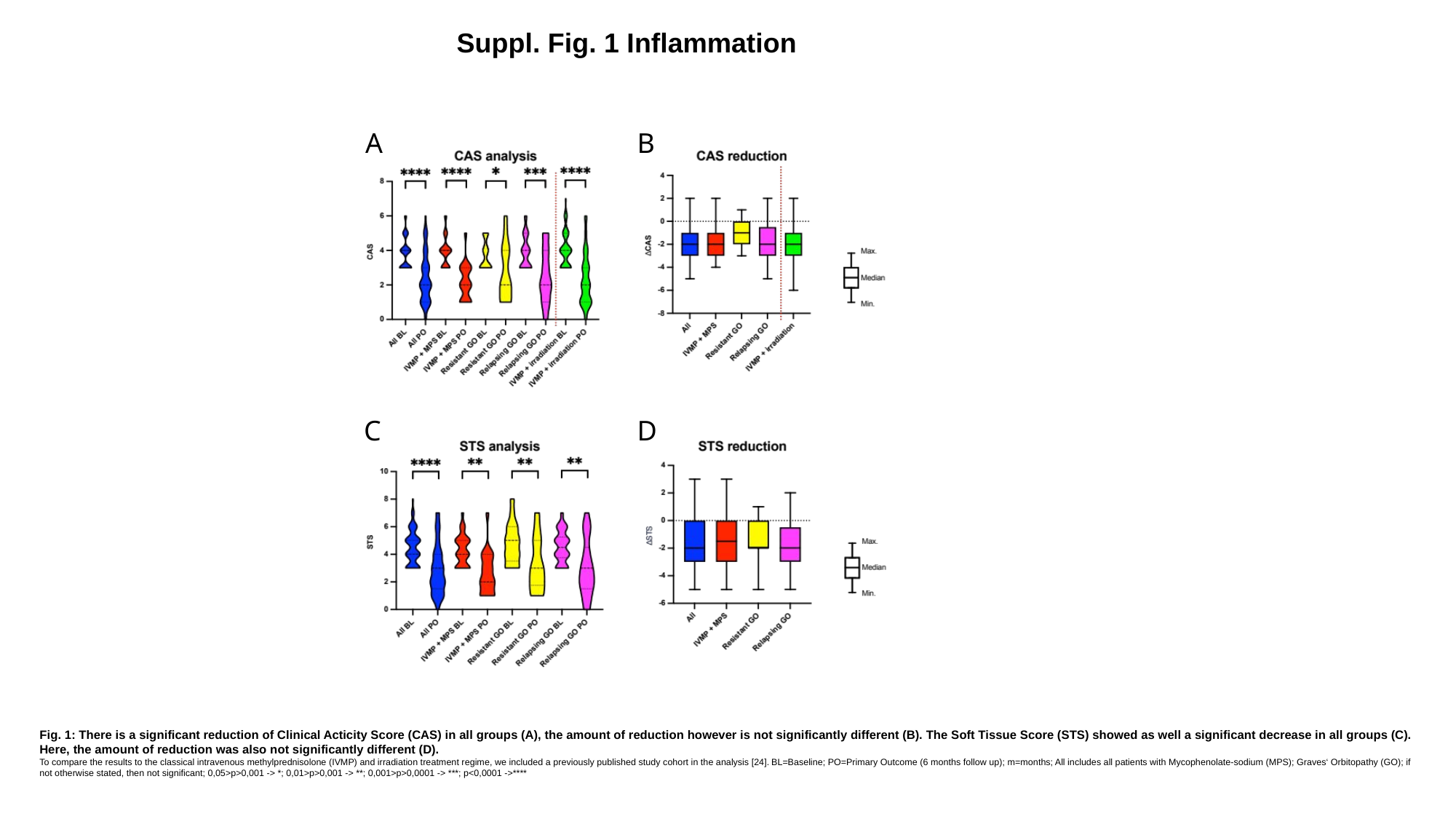

Suppl. Fig. 1 Inflammation
B
A
C
D
Fig. 1: There is a significant reduction of Clinical Acticity Score (CAS) in all groups (A), the amount of reduction however is not significantly different (B). The Soft Tissue Score (STS) showed as well a significant decrease in all groups (C). Here, the amount of reduction was also not significantly different (D).
To compare the results to the classical intravenous methylprednisolone (IVMP) and irradiation treatment regime, we included a previously published study cohort in the analysis [24]. BL=Baseline; PO=Primary Outcome (6 months follow up); m=months; All includes all patients with Mycophenolate-sodium (MPS); Graves‘ Orbitopathy (GO); if not otherwise stated, then not significant; 0,05>p>0,001 -> *; 0,01>p>0,001 -> **; 0,001>p>0,0001 -> ***; p<0,0001 ->****

## Slide 2
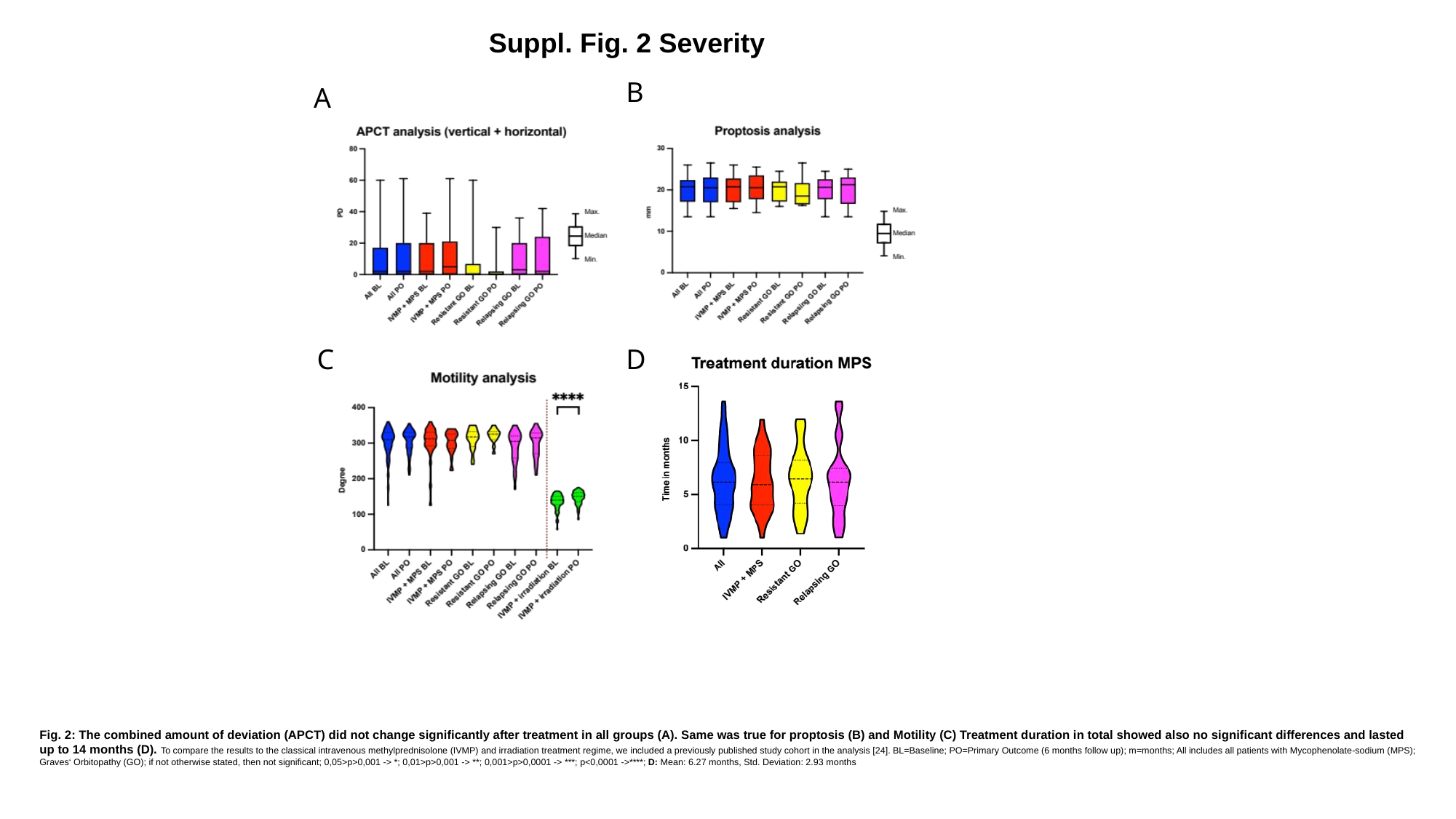

Suppl. Fig. 2 Severity
B
A
C
D
Fig. 2: The combined amount of deviation (APCT) did not change significantly after treatment in all groups (A). Same was true for proptosis (B) and Motility (C) Treatment duration in total showed also no significant differences and lasted up to 14 months (D). To compare the results to the classical intravenous methylprednisolone (IVMP) and irradiation treatment regime, we included a previously published study cohort in the analysis [24]. BL=Baseline; PO=Primary Outcome (6 months follow up); m=months; All includes all patients with Mycophenolate-sodium (MPS); Graves‘ Orbitopathy (GO); if not otherwise stated, then not significant; 0,05>p>0,001 -> *; 0,01>p>0,001 -> **; 0,001>p>0,0001 -> ***; p<0,0001 ->****; D: Mean: 6.27 months, Std. Deviation: 2.93 months

## Slide 3
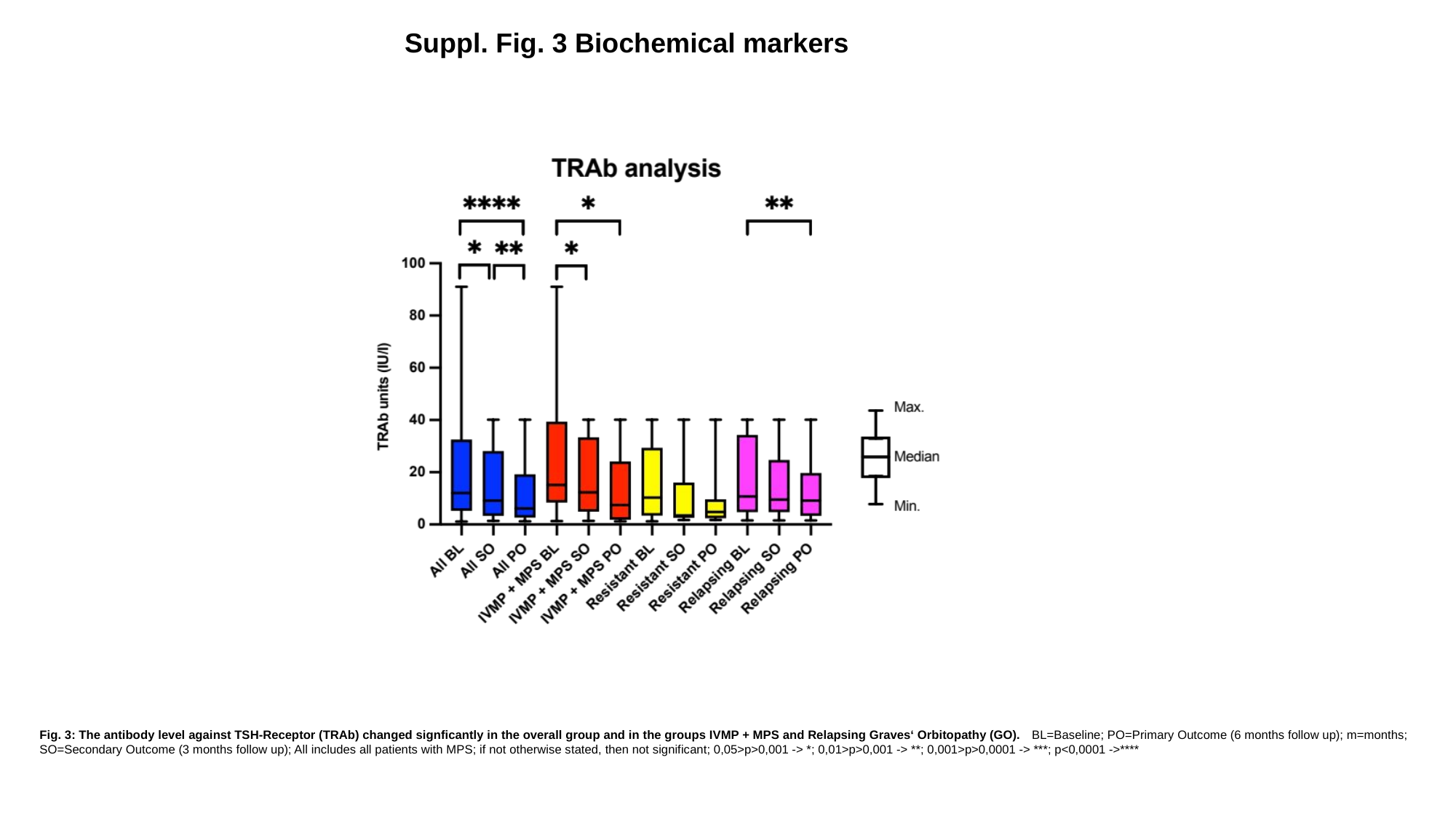

Suppl. Fig. 3 Biochemical markers
Fig. 3: The antibody level against TSH-Receptor (TRAb) changed signficantly in the overall group and in the groups IVMP + MPS and Relapsing Graves‘ Orbitopathy (GO).   BL=Baseline; PO=Primary Outcome (6 months follow up); m=months; SO=Secondary Outcome (3 months follow up); All includes all patients with MPS; if not otherwise stated, then not significant; 0,05>p>0,001 -> *; 0,01>p>0,001 -> **; 0,001>p>0,0001 -> ***; p<0,0001 ->****
